# Coronary microvascular function and visceral adiposity in patients with normal body weight and type 2 diabetes

**DOI:** 10.1002/oby.23413

**Published:** 2022-03-31

**Authors:** Amrit Chowdhary, Sharmaine Thirunavukarasu, Nicholas Jex, Lauren Coles, Charles Bowers, Anshuman Sengupta, Peter Swoboda, Klaus Witte, Richard Cubbon, Hui Xue, Peter Kellman, John Greenwood, Sven Plein, Eylem Levelt

**Affiliations:** ^1^ Multidisciplinary Cardiovascular Research Centre and Biomedical Imaging Science Department Leeds Institute of Cardiovascular and Metabolic Medicine University of Leeds Leeds UK; ^2^ Discovery and Translational Science Department Leeds Institute of Cardiovascular and Metabolic Medicine University of Leeds Leeds UK; ^3^ National Heart, Lung, and Blood Institute National Institutes of Health Department of Health and Human Services Bethesda Maryland USA

## Abstract

**Objective:**

This study sought to assess whether diabetes affects coronary microvascular function in individuals with normal body weight.

**Methods:**

Seventy‐five participants (30 patients with type 2 diabetes [T2D] who were overweight [O‐T2D], 15 patients with T2D who were lean [LnT2D], 15 healthy volunteers who were lean [LnHV], and 15 healthy volunteers who were overweight [O‐HV]) without established cardiovascular disease were recruited. Participants underwent magnetic resonance imaging for assessment of subcutaneous, epicardial, and visceral adipose tissue areas, adenosine stress myocardial blood flow (MBF), and cardiac structure and function.

**Results:**

Stress MBF was reduced only in the O‐T2D group (mean [SD], LnHV = 2.07 [0.47] mL/g/min, O‐HV = 2.08 [0.42] mL/g/min, LnT2D = 2.16 [0.36] mL/g/min, O‐T2D = 1.60 [0.28] mL/g/min; *p* ≤ 0.0001). Accumulation of visceral fat was evident in the LnT2D group at similar levels to the O‐HV group (LnHV = 127 [53] cm^2^, O‐HV = 181 [60] cm^2^, LnT2D = 182 [99] cm^2^, O‐T2D = 288 [72] cm^2^; *p* < 0.0001). Only the O‐T2D group showed reductions in left ventricular ejection fraction (LnHV = 63% [4%], O‐HV = 63% [4%], LnT2D = 60% [5%], O‐T2D = 58% [6%]; *p* = 0.0008) and global longitudinal strain (LnHV = −15.1% [3.1%], O‐HV= −15.2% [3.7%], LnT2D = −13.4% [2.7%], O‐T2D = −11.1% [2.8%]; *p* = 0.002) compared with both control groups.

**Conclusions:**

Patients with T2D and normal body weight do not show alterations in global stress MBF, but they do show significant increases in visceral adiposity. Patients with T2D who were overweight and had no prior cardiovascular disease showed an increase in visceral adiposity and significant reductions in stress MBF.

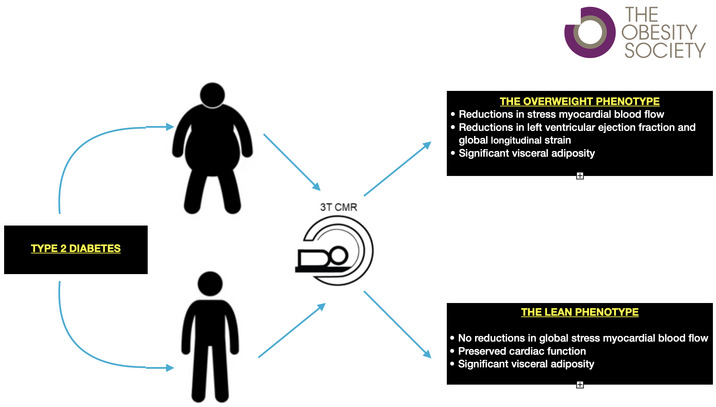

## Graphical abstract

Changes in cardiac structure, function, perfusion and adipose tissue distribution between lean and overweight patients with T2D and HV.

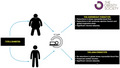

**Study Importance**

**What is already known?**

► The prevalence of type 2 diabetes (T2D) is on the rise, fueled at least partly by the obesity pandemic, with heart failure as one of its major cardiovascular complications.
► A total of 9% to 21% of adults have normal body weight at the time of diabetes diagnosis.
► Coronary microvascular dysfunction (CMD) has emerged as a potential causative mechanism for development of heart failure.
► CMD has been demonstrated in obesity. Whether diabetes affects CMD in the absence of obesity remains unknown.

**What does this study add?**

► Patients with T2D and normal body weight, with no prior cardiovascular disease, do not show alterations in global stress myocardial blood flow or in cardiac function and strain parameters.
► Accumulation of visceral fat is evident even in patients with T2D and normal body weight.
► Patients with T2D and overweight show significantly higher visceral fat accumulation compared with BMI‐matching individuals without diabetes or overweight.
► Reductions in global stress myocardial blood flow are evident only in patients with diabetes and overweight and with no prior cardiovascular disease.

**How might these results change the direction of research?**

► Future studies are needed to further characterize the adipose tissue remodeling and function in patients with T2D and the role adipose tissue plays in the development of cardiac dysfunction in diabetes.

## INTRODUCTION

Driven predominantly by the obesity epidemic, the prevalence of type 2 diabetes (T2D) continues to rise (1), with heart failure as the leading cardiovascular complication (2) even in patients with good cardiovascular risk factor management (3). Coronary microvascular dysfunction (CMD) has emerged as a candidate mechanism of heart disease in diabetes (4, 5), preceding clinical heart failure manifestation (6, 7) and carrying important prognostic information (8, 9). However, CMD has also been demonstrated in individuals with obesity and without diabetes (10, 11). The proportion of adults with normal body weight at the time of incident diabetes has ranged from 9% to 21% (12, 13). Whether or not diabetes affects coronary microvascular function in the absence of obesity is uncertain.

Moreover, individuals with the same BMI do not necessarily share the same degree of adiposity and they may have a very different fat mass percentage and fat distribution, with diverse metabolic consequences (14). It has been proposed that the visceral adipose tissue (VAT), including the epicardial adipose tissue (EAT), may have a more adverse influence on cardiovascular health compared with subcutaneous adipose tissue (SAT), as it is a metabolically active tissue capable of secreting adipokines and proinflammatory mediators that regulate appetite and insulin action (15, 16). Moreover, highlighting the cardiometabolic relevance of distinct adipose tissue distribution phenotypes, a lower amount of lower‐body fat mass has been shown to be an important determinant of cardiometabolic risk (17, 18). Novel findings have suggested an important and independent role of increased gluteofemoral fat mass to maintain metabolic health (19). Although the role of excess visceral adiposity in individuals with normal body weight has been carefully studied in the context of prediabetes (20, 21), there is sparse evidence to show whether patients with diabetes and normal body weight possess excess visceral adiposity compared with their weight‐matching counterparts with no diabetes, as well as whether diabetes and obesity have a combined impact on the alterations of adipose tissue distribution.

First‐pass dynamic contrast‐enhanced myocardial perfusion cardiovascular magnetic resonance imaging (CMR) can be used to derive quantitative estimates of hyperemic and resting myocardial blood flow (MBF) for assessment of myocardial microvascular function (22). CMR is also the reference technique for a comprehensive, noninvasive assessment of changes in cardiac structure, function, strain, fibrosis, and scar (23). VAT, SAT, and EAT can be measured with high accuracy by magnetic resonance imaging (MRI) (24).

Consequently, using CMR, we sought to establish whether diabetes affects coronary microvascular function in patients with T2D and normal body weight (LnT2D). We further aimed to compare adipose tissue distribution and cardiac structural and functional alterations between the asymptomatic LnT2D group and patients who were overweight with T2D (O‐T2D) and without established cardiovascular disease, as well as between healthy volunteers who were lean and those who were overweight (LnHV and O‐HV, respectively).

## METHODS

### Study population and design

This single‐center observational study was approved by the National Research Ethics Committee (REC Ref 18/YH/0168) and was conducted in accordance with the Declaration of Helsinki. Informed written consent was obtained from each participant. A total of 30 participants in the O‐T2D group (BMI > 25 kg/m^2^), 15 participants in the LnT2D group (BMI ≤ 25), 15 participants with no T2D in the LnHV group (BMI ≤ 25), and 15 participants with no T2D in the O‐HV group (BMI > 25), all with a similar age and sex distribution, were recruited to the study. Patients were recruited from the general practices in Yorkshire, UK. Control participants were recruited from local golf clubs.

### Inclusion and exclusion criteria

Participants were excluded if they had a previous diagnosis of cardiovascular disease (previous coronary artery bypass graft surgery, angioplasty, myocardial infarction, angina, moderate or above valvular heart disease, and atrial fibrillation), contraindications to CMR, ischemic changes on 12‐lead electrocardiogram (ECG), or renal impairment (estimated glomerular filtration rate below 30 mL/min/1.73 m^2^) or if they were receiving treatment with insulin. Control participants had no overt cardiovascular disease, and they had normal glycemic control with glycated hemoglobin (HbA1c) values ≤40 mmol/mol. For the control cohorts, at the point of recruitment, it was ascertained by verbal questioning that the exercise duration had been <6 h/wk for the past 12 months.

### Anthropometric measurements

Height and weight were recorded, BMI was calculated, blood pressure (BP) was recorded as an average of three supine measures taken over 10 minutes (DINAMAP‐1846‐SX, Critikon Inc., Tampa, Florida), and a resting ECG was recorded. Fasting blood samples were taken from each participant for assessments of full blood count, estimated glomerular filtration rate, glucose, insulin, HbA1c, triglycerides, high‐density lipoprotein (HDL), low‐density lipoprotein (LDL), and total cholesterol levels. Homeostasis model assessment of insulin resistance index (HOMA‐IR; fasting serum insulin [micrometers per liter] × fasting plasma glucose [millimoles per liter]/22.5) (14, 25) and triglyceride to HDL ratio (26) were calculated as validated surrogate markers of insulin resistance from fasting blood samples.

### MRI

**FIGURE 1:**
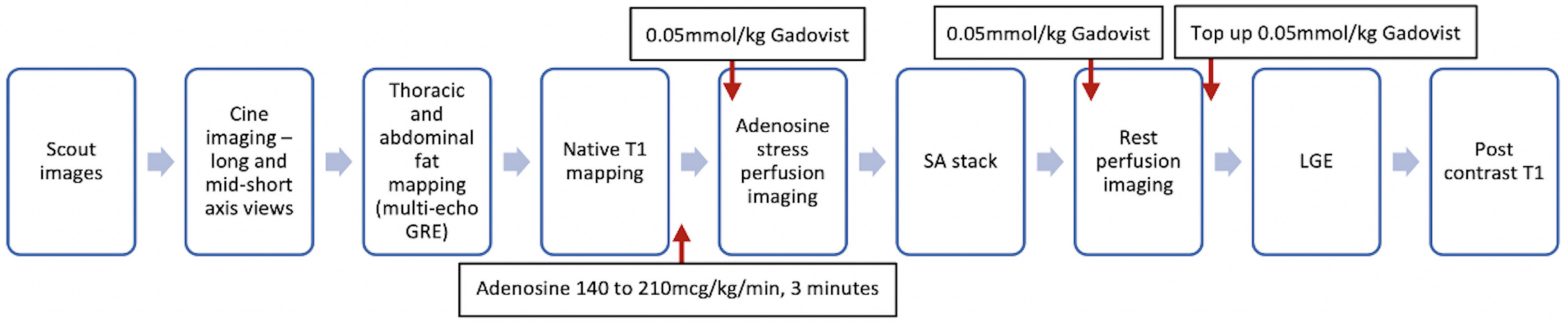
Scan protocol. The scan protocol included cine imaging, thoracic and abdominal fat/water maps, native precontrast and postcontrast T1 mapping, adenosine stress perfusion imaging, and LGE imaging. SA, short axis; LGE, late gadolinium enhancement; GRE, gradient echo [Color figure can be viewed at wileyonlinelibrary.com]

All scans were performed on a 3.0‐T MR system (Prisma, Siemens, AG, Erlangen, Germany). Participants were advised to avoid caffeine for 24 hours prior. The MRI protocol (Figure 1) consisted of cine imaging using a steady‐state free precession (SSFP) sequence, thoracic and abdominal water/fat images using a multi‐echo gradient echo (GRE) sequence, native and postcontrast T1 mapping, stress and rest perfusion, and late gadolinium enhancement (LGE).

For epicardial and abdominal visceral fat area measurements, single‐shot acquisition of thoracic and abdominal non‐breath‐hold images was performed using a multi‐echo GRE sequence with gradient flyback for monopolar readout to acquire three echoes for each phase encode (parallel imaging rate 3 using GRAPPA with separate reference line acquisition, 32‐channel cardiac array; bandwidth = 1,184 Hz/pixel; echo time (TE) = 1.33, 3.35, and 5.37 milliseconds; repetition time (TR) = 6.71 milliseconds; readout flip angle = 20°; matrix = 192 × 108; single‐shot duration = 242 milliseconds; field of view (FOV)= 300 × 225 mm^2^; section thickness = 6 mm), as previously described (27).

Native T1 mapping was acquired in three sections using a breath‐held modified Look‐Locker inversion recovery acquisition, as previously described (precontrast 5 seconds [3 seconds] 3 seconds and postcontrast 4 seconds [1 second] 3 seconds [1 second] 2 seconds schemes) (28). Postcontrast T1 mapping acquisition was performed 15 minutes after the last contrast injection using identical planning as the native T1 map.

Perfusion imaging used a free‐breathing, fast low‐angle shot (FLASH) MR protocol with motion‐corrected (MOCO) automated in‐line perfusion mapping using the Gadgetron streaming software image reconstruction framework, as previously described (23). For stress perfusion imaging, adenosine was infused at a rate of 140 µg/kg/min and increased up to a maximum of 210 µg/kg/min according to hemodynamic and symptomatic response (a significant hemodynamic response to adenosine stress was defined as a >10‐beats/min increase in heart rate or a BP drop < 10 mm Hg and >1 adenosine‐related symptom, e.g., chest tightness, breathlessness) (29). A minimum 10‐minute interval was kept between perfusion acquisitions to ensure equilibration of gadolinium kinetics and resolution of all hemodynamic effects of adenosine. For each perfusion acquisition, an intravenous bolus of 0.05 mmol/kg of gadobutrol (Gadovist, Leverkusen, Germany) was administered at 5 mL/s followed by a 20‐mL saline flush using an automated injection pump (Medrad MRXperion Injection System, Bayer, Leverkusen, Germany).

LGE imaging was performed using a phase‐sensitive inversion recovery sequence in matching left ventricle (LV) short‐axis planes and long‐axis planes >8 minutes after contrast administration to exclude the presence of previous myocardial infarction or regional fibrosis.

### Quantitative analysis

All CMR postprocessing analysis was performed offline and blinded to all participant details by AC (with 2 years of CMR experience) after completion of the study. The anonymization codes, which were generated using a random number generator, were unlocked only after all data analysis was completed.

All CMR image analysis was performed offline and blinded to all participant details by AC, and all scan contours were subsequently reviewed using cvi42 software (Circle Cardiovascular Imaging, Calgary, Canada) by EL (with 8 years of CMR experience; level 3 European Association of Cardiovascular Imaging accreditation), who was also blinded to participant details. Images for biventricular volumes and function were analyzed as previously described (30). The left atrial (LA) volume and LA ejection fraction were calculated using the biplane area‐length method in the horizontal and vertical long axes, as previously described (31). Strain measurements were performed using cvi42 Tissue Tracking (Circle Cardiovascular Imaging) from balanced SSFP from the short‐axis images and the horizontal long‐axis and vertical long‐axis views. The peak circumferential systolic strain and peak early diastolic strain rates and global longitudinal strain (GLS) were measured as previously described (32).

Myocardial perfusion image reconstruction and processing were implemented using the Gadgetron software framework as previously described (23). Rest/stress MBF was measured for each of the 16 segments using the American Heart Association (AHA) classification. MBF values for all remaining segments were averaged to provide a global value. Native T1 maps and extracellular volume (ECV) were analyzed using cvi42 software from a region of interest in the midwall of the septum using the native precontrast and native postcontrast T1 times of myocardium, blood pool, and hematocrit, as previously described (33). Myocardial cell volume was calculated from native T1 maps by using the following calculation: (left ventricular mass/1.05) × (1 − ECV), as previously described (34).

**FIGURE 2:**
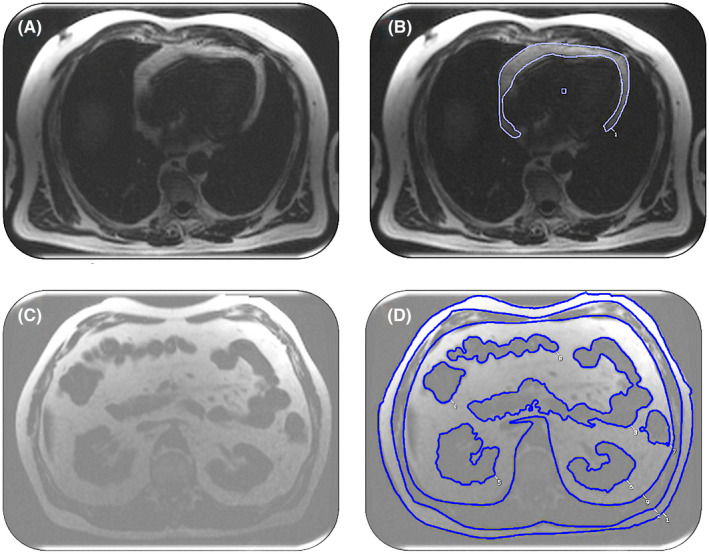
Epicardial, visceral, and subcutaneous adipose tissue imaging and analysis. (A) Multi‐echo GRE sequence image acquired on transaxial orientation of a four‐chamber view on a single section showing epicardial adipose tissue. (B) Representative example of contouring of epicardial adipose tissue on a single‐section four‐chamber view. (C) Multi‐echo GRE sequence abdominal imaging used to characterize adipose tissue into visceral and subcutaneous fat through manual division. (D) Representative example of manual contouring and segmentation of the abdominal adipose tissue into subcutaneous and visceral components. GRE, gradient echo [Color figure can be viewed at wileyonlinelibrary.com]

Abdominal VAT area was measured at the level of the third to fourth lumbar vertebral body from the single shot performed using multi‐echo GRE sequence abdominal images. Adipose tissue was categorized into VAT and SAT through manual division, which was accomplished by drawing a line following the abdominal wall to separate intra‐ and extra‐abdominal compartments. The EAT area was traced from the thoracic multi‐echo GRE sequence images acquired on transaxial orientation of a four‐chamber view on a single section (Figure 2A,B). The VAT and SAT areas were segmented separately using cvi42 software (Figure 2C,D), and VAT over SAT area was calculated.

### Qualitative perfusion and scar assessment

The CMR perfusion images were interpreted visually by EL. Rest/stress perfusion images were carefully reviewed for each of the 16 segments using the AHA classification.

For LGE imaging analysis, areas of contrast enhancement were visually scored as absent or present by two operators (AC and EL). Hyperenhancement was considered present only if myocardial enhancement was confirmed on both short‐axis and perpendicular long‐axis locations.

### Statistical analysis

Statistical analysis was performed using SPSS Statistics version 26.0 (IBM Corp., Armonk, New York). Categorical data were compared with the Pearson χ^2^ test. Continuous variables are presented as mean (SD) and they were checked for normality using the Shapiro–Wilk test. Comparisons between the four groups were performed by one‐way ANOVA with post hoc Bonferroni corrections. Based on the Bonferroni correction, considering the six comparisons between the four groups, *p* < 0.003 was applied as indicating statistical significance.

The Student *t* test was used for comparison of normally distributed data sets in which data were obtained for only the LnT2D and O‐T2D groups. Bivariate correlations were performed using the Pearson correlation coefficient. For these tests, *p* ≤ 0.05 was considered statistically significant.

Sample size calculations were performed before the study (T2D = 1.74 [0.24] mL/g/min, controls = 2.12 [0.26] mL/g/min), which suggested that, to detect a 25% difference in stress MBF across the four cohorts, 12 participants per group would be needed (with 80% power at *α* = 0.05). There was no difference in rest MBF data between patients with T2D and control patients with no diabetes. In line with the higher prevalence of diabetes and overweight/obesity comorbidity compared with T2D in isolation in the general population, there were more volunteers with T2D and overweight who expressed interest in participating in the study. All eligible participants who expressed interest in the study during the predetermined recruitment phase were included. Consequently, more participants were recruited into the O‐T2D group, whereas the other three groups were matched in sample size. Overall recruitment goals were exceeded in the study; 15 participants were recruited to the LnT2D, LnHV, and O‐HV groups each, and 30 participants were recruited to the O‐T2D group.

## RESULTS

### Participant demographics and biochemical characteristics

Demographic, clinical, and biochemical data of the four study groups are shown in Table 1.

**TABLE 1:** Demographics, biochemical characteristics, and medications

	LnHV (*n* = 15)	O‐HV (*n* = 15)	LnT2D (*n* = 15)	O‐T2D (*n* = 30)	ANOVA/χ^2^
Age (y)	63 ± 7	66 ± 8	63 ± 12	65 ± 11	0.8
Sex (M), *n* (%)	10 (67)	8 (53)	9 (60)	23 (69)	0.3
Duration of diabetes (y)	–	–	13 ± 6	11 ± 3	0.2
Systolic BP (mm Hg)	130 ± 13	133 ± 13	126 ± 17	132 ± 14	0.6
Diastolic BP (mm Hg)	77 ± 7	76 ± 7	72 ± 8	76 ± 6	0.08
Heart rate (bpm)	63 ± 10	67 ± 11	69 ± 13	67 ± 10	0.7
Height (cm)	171 ± 9	169 ± 8	168 ± 10	169 ± 10	0.5
Weight (kg)	70 ± 10^a^, ^b^	83 ± 8^a^	68 ± 10	86 ± 10^b^	**<0.0001**
BMI (kg/m^2^)	23 ± 2^a^, ^c^	29 ± 2^d^	23 ± 1^b^	30 ± 3	**<0.0001**
Waist circumference (cm)	90 ± 7^a^, ^c^	105 ± 6^d^	92 ± 11^b^	109 ± 8	**<0.0001**
Waist‐hip ratio	0.95 ± 0.08^a^, ^c^	1.01 ± 0.06^d^	0.94 ± 0.10^b^	1.01 ± 0.05	**0.0001**
Hemoglobin (g/L)	141 ± 9	148 ± 8	140 ± 11	149 ± 17	0.07
Creatinine (μmol/L)	71 ± 11	71 ± 11	64 ± 14	73 ± 17	0.3
eGFR (mL/min/1.73 m^2^)	82 ± 8	81 ± 6	87 ± 7	82 ± 11	0.5
Total cholesterol (mmol/L)	5.85 ± 0.65^c^	5.17 ± 1.35	4.71 ± 1.19	4.72 ± 1.22	0.04
HDL (mmol/L)	1.94 ± 0.58^a^, ^c^, ^e^	1.53 ± 0.38	1.61 ± 0.45^b^	1.26 ± 0.22	**<0.0001**
LDL (mmol/L)	3.35 ± 0.38	2.99 ± 1.10	2.41 ± 0.97	2.73 ± 1.31	0.1
TG (mmol/L)	1.22 ± 0.65^c^	1.49 ± 0.64	1.60 ± 0.82	2.50 ± 1.57	**0.002**
Fasting glucose (mmol/L)	4.9 ± 0.6^c^, ^e^	5.2 ± 0.4^d^, ^f^	8.7 ± 2.9	9.3 ± 3.9	**0.0002**
HbA1c (mmol/mol)	37 ± 3^c^, ^e^	38 ± 2^d^, ^f^	58 ± 8	63 ± 19	**<0.0001**
Insulin (pmol/L)	25 ± 15^c^	67 ± 35^f^	41 ± 29^b^	173 ± 115	**<0.0001**
C peptide (pmol/L)	432 ± 205^c^	745 ± 300	553 ± 265	945 ± 695	0.01
HOMA‐IR	0.79 ± 0.49^c^	2.21 ± 1.23^f^	2.40 ± 2.02^b^	10.21 ± 6.99	**<0.0001**
TG‐HDL ratio	0.76 ± 0.63^c^	1.05 ± 0.48^f^	1.14 ± 0.84^b^	2.38 ± 1.75	**0.002**
Medications, *n* (%)					
ACE inhibitor			4 (20)	14 (47)	0.2
ARB			3 (20)	5 (17)	0.1
β blocker			0 (0)	5 (17)	**0.02**
Calcium channel blocker			3 (20)	4 (13)	0.6
Aspirin			6 (40)	7 (23)	0.6
Statin			10 (67)	22 (73)	0.1
Metformin			10 (67)	15 (50)	0.3
Sulphonylurea			4 (27)	8 (27)	1.0
GLP‐1RA			0 (0)	0 (0)	‐
Gliptins			4 (27)	6 (20)	0.6
SGLT2i			1 (6)	2 (7)	0.4
Thiazolidinediones			3 (20)	1 (3)	0.06

Note

Values are mean ± SD for continuous variables and number (%) for categorical variables. Values in bold signify statistical significance.

Abbreviations: ACEI, angiotensin converting enzyme; ARB, angiotensin receptor blocker; BP, blood pressure; bpm, beats per minute; eGFR, estimated glomerular filtration rate; GLP‐1RA, glucagon‐like peptide 1 receptor agonist; HbA1c, glycated hemoglobin; HDL, high‐density lipoprotein; HOMA‐IR, homeostasis model assessment of insulin resistance index; HV, healthy volunteers; LDL, low‐density lipoprotein; LnHV, lean HV; LnT2D, lean patients with type 2 diabetes; O‐HV, HV with overweight; O‐T2D, patients with overweight and type 2 diabetes; SGLT2i, sodium glucose contransporter‐2 inhibitors; TG, triglyceride.

^a^ *p* < 0.05 between the LnHV and O‐HV groups.

^b^ *p* < 0.05 between the LnT2D and O‐T2D groups.

^c^ *p* ≤ 0.05 between the LnHV and O‐T2D groups.

^d^ *p* ≤ 0.05 between the O‐HV and LnT2D groups.

^e^ *p* < 0.05 between the LnHV and LnT2D groups.

^f^ *p* < 0.05 between the O‐HV and O‐T2D groups.

A total of 30 participants in the O‐T2D group (mean [SD], age = 65 [11] years, BMI = 30 [3], HbA1c = 63 [19] mmol/mol), 15 in the LnT2D group (age = 63 [12] years, BMI = 23 [1], HbA1c = 58 [8] mmol/mol), 15 in the LnHV group (age = 63 [7] years, BMI = 23 [2], HbA1c = 37 [3] mmol/mol), and 15 in the O‐HV group (age = 66 [8] years, BMI = 29 [2], HbA1c = 38 [2] mmol/mol) were recruited. Age and sex distribution were similar between the cohorts. The two T2D groups were matched for diabetes treatment and duration. The two lean groups and the two groups with overweight were matched for BMI. Control patients were not receiving any medications.

There was no significant difference in systolic BP or heart rate across the groups. Waist circumference and waist‐hip circumference ratio were similarly increased in both groups with overweight (O‐T2D and O‐HV) compared with the lean groups (LnT2D and LnHV). Plasma triglyceride and insulin levels, HOMA‐IR calculations, and triglyceride‐HDL ratio were higher in the O‐T2D group compared with the other three cohorts.

### Multiparametric MRI results

CMR results for cardiac volumes and function, perfusion, native T1 maps, ECV, and thoracic and abdominal MRI results for EAT, VAT, and SAT areas are summarized in Table 2.

**TABLE 2:** MRI parameters

	LnHV (*n* = 15)	O‐HV (*n* = 15)	LnT2D (*n* = 15)	O‐T2D (*n* = 30)	ANOVA
LV end diastolic volume (mL)	152 ± 33	140 ± 30	125 ± 28	143 ± 29	0.09
LV end diastolic volume index (mL/m^2^)	82 ± 13	72 ± 13	70 ± 12	72 ± 15	0.06
LV end systolic volume (mL)	56 ± 13	52 ± 16	50 ± 14^a^	61 ± 17	0.1
LV end systolic volume index (mL/m^2^)	31 ± 6	27 ± 7	29 ± 7	30 ± 8	0.3
LV stroke volume (mL)	95 ± 22	88 ± 16	75 ± 17	82 ± 16	0.01
LV ejection fraction (%)	63 ± 4^b^	63 ± 4^c^	60 ± 5	58 ± 6	**0.0008**
LV mass (g)	93 ± 27	95 ± 29	79 ± 19	96 ± 20	0.1
LV mass index (g/m^2^)	52 ± 12	52 ± 11	48 ± 10	48 ± 10	0.2
LV mass/LV end diastolic volume (mg/mL)	0.65 ± 0.12	0.72 ± 0.15	0.69 ± 0.21	0.68 ± 0.14	0.5
RV end diastolic volume (mL)	161 ± 36	153 ± 43	122 ± 30	142 ± 30	0.02
RV end diastolic volume index (mL/m^2^)	87 ± 14^b^, ^d^	78 ± 18	69 ± 12	70 ± 14	**0.001**
RV end systolic volume (mL)	67 ± 22	61 ± 24	54 ± 17	63 ± 17	0.6
RV stroke volume (mL)	93 ± 19	92 ± 21	69 ± 17	79 ± 18	0.7
RV ejection fraction (%)	60 ± 7	60 ± 5	56 ± 6	56 ± 6	0.06
Native T1 (ms)	1207 ± 81	1166 ± 84	1148 ± 111	1197 ± 69	0.09
Extracellular volume (%)	23 ± 3	22 + 3	23 ± 2	22 ± 3	0.5
Cell volume (mL/m^2^)	70 ± 18	74 ± 17	64 ± 20	73 ± 17	0.8
Peak circumferential strain, negative (%)	21.3 ± 2.7^b^	21.9 ± 2.2^c^	21.7 ± 3.3^a^	18.3 ± 3.0	**0.0005**
GLS, negative (%)	15.1 ± 3.1^b^	15.2 ± 3.7^c^	13.4 ± 2.7	11.1 ± 2.8	**0.002**
Peak diastolic strain rate, (L/s)	1.18 ± 0.25	1.15 ± 0.18	1.22 ± 0.29^a^	1.00 ± 0.21	0.01
LA maximum volume (mL)	57 ± 24	61 ± 20	61 ± 16	66 ± 24	0.6
LA maximum volume indexed (mL/m^2^)	33 ± 13	32 ± 11	34 ± 8	31 ± 12	0.8
LA ejection fraction (%)	57 ± 9	63 ± 13	55 ± 8	54 ± 11	0.09
Myocardial perfusion					
Stress MBF (mL/g/min)	2.07 ± 0.47^b^	2.08 ± 0.42^c^	2.16 ± 0.36^a^	1.60 ± 0.28	**<0.0001**
Rest MBF (mL/g/min)	0.64 ± 0.08	0.72 ± 0.15	0.74 ± 0.13	0.67 ± 0.15	0.1
Myocardial perfusion reserve index	3.18 ± 0.84	3.17 ± 0.59	2.98 ± 0.66	2.47 ± 0.62	0.009
Stress RPP (bpm × mm Hg)	11,942 ± 3256	11,764 ± 3102	11,464 ± 2100	11,672 ± 2515	0.7
Rest RPP (bpm × mm Hg)	8339 ± 1585	8265 ± 1723	8645 ± 1599	8934 ± 1902	0.5
Increase in RPP (%)	39	38	37	32	0.7
Adipose tissue measurements					
SAT (cm^2^)	106 ± 31	169 ± 55	147 ± 71	171 ± 80	0.06
VAT (cm^2^)	127 ± 53^b^	181 ± 60^c^	182 ± 99^a^	288 ± 72	**<0.0001**
EAT (cm^2^)	14 ± 6^b^	17 ± 5^c^	18 ± 8^a^	31 ± 13	**<0.0001**
VAT/SAT	1.23 ± 0.43^b^	1.15 ± 0.49^c^	1.56 ± 1.24^a^	2.05 ± 1.00	**0.0007**

Note

Values are mean ± SD for continuous variables and number (%) for categorical variables. Values in bold signify *p* < 0.05.

Abbreviations: bpm, beats per min; EAT, epicardial adipose tissue; GLS, global longitudinal strain; HV, healthy volunteers; LA, left atrial; LnHV, lean HV; LnT2D, lean patients with type 2 diabetes; LV, left ventricular; MBF, myocardial blood flow; O‐HV, HV with obesity/overweight; O‐T2D, patients with overweight and type 2 diabetes; RPP, rate pressure product; RV, right ventricular; SAT, subcutaneous adipose tissue; VAT, visceral adipose tissue.

^a^ *p* < 0.05 between the LnT2D and O‐T2D groups.

^b^ *p* ≤ 0.05 between the LnHV and O‐T2D groups.

^c^ *p* < 0.05 between the O‐HV and O‐T2D groups.

^d^ *p* < 0.05 between the LnHV and LnT2D groups.

#### Cardiac geometry and function

Only patients in the O‐T2D group showed reductions in LV ejection fraction compared with both control groups (LnHV = 63% [4%], O‐HV = 63% [4%], LnT2D = 60% [5%], O‐T2D = 58% [6%]; *p* = 0.0008). Although significant reductions in peak circumferential systolic strain and GLS were detected in the O‐T2D group compared with both the weight‐matching and the lean control groups, peak circumferential systolic strain was not reduced in patients in the LnT2D group. The numeric differences in peak diastolic strain rate (PDSR) across the groups did not reach statistical significance.

The LnT2D group had the lowest LV end diastolic volume, mass, and myocardial cell volume compared with the other three groups, and there was no significant difference in ECV between the four groups.

There was no difference in LA volume or function across the groups.

#### MBF

Rest and stress rate pressure product values, MBF, and myocardial perfusion reserve (MPR) measurements are summarized in Table 2. Participants from all groups demonstrated a similar increase in rest and stress rate pressure products during adenosine stress.

**FIGURE 3:**
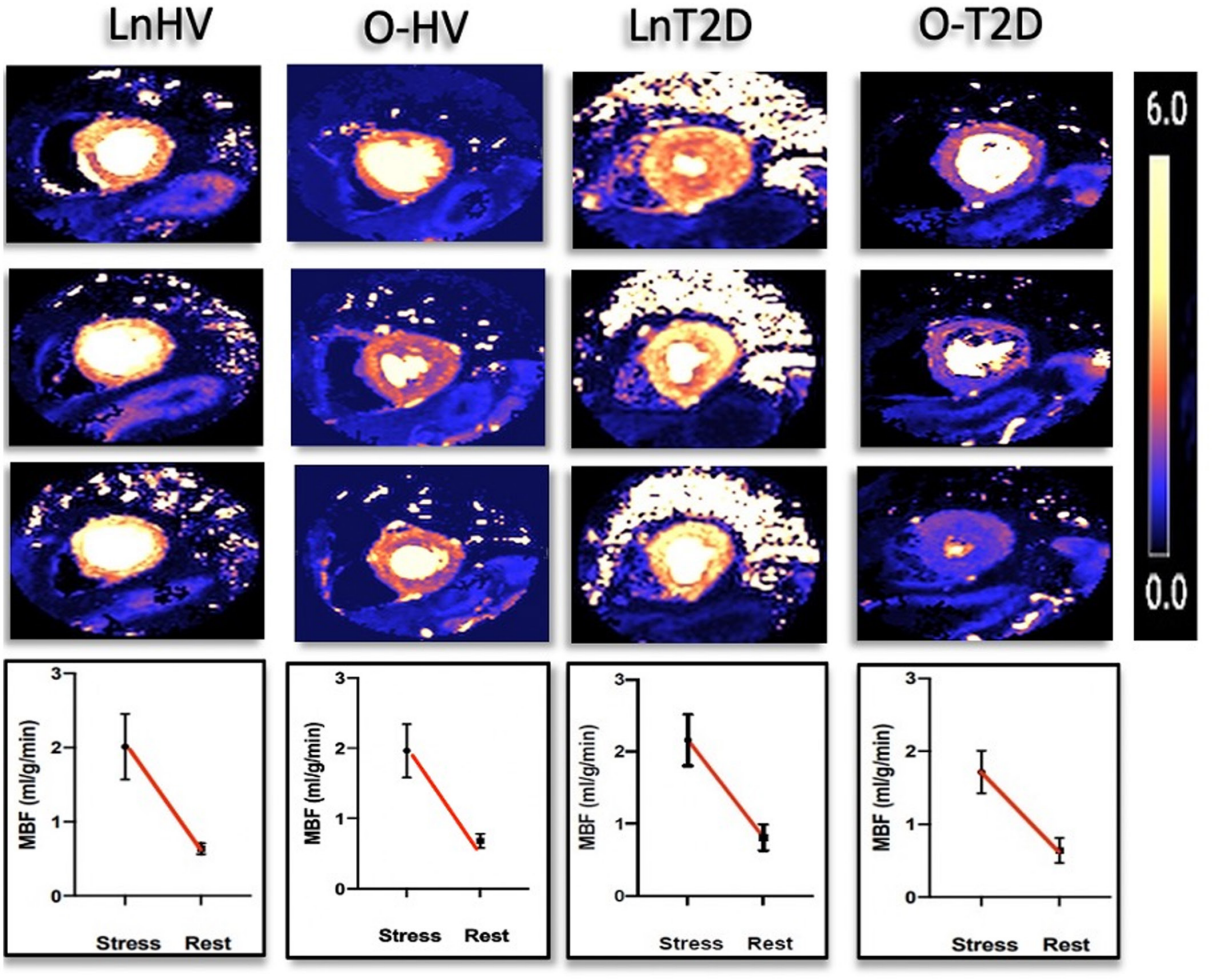
Representative MBF maps acquired at basal, midventricular, and apical levels during peak stress, along with graphs showing the changes in MBF from peak stress to rest in lean healthy volunteers and healthy volunteers with overweight, as well as lean patients with T2D and patients with overweight and T2D. MBF, myocardial blood flow; T2D, type 2 diabetes; HV, healthy volunteers; LnHV, lean HV; LnT2D, lean patients with type 2 diabetes; O‐HV, HV with overweight; O‐T2D, patients with overweight and type 2 diabetes [Color figure can be viewed at wileyonlinelibrary.com]

Only the O‐T2D group showed significant reductions in stress MBF (LnHV = 2.07 [0.47] mL/g/min, O‐HV = 2.08 [0.42] mL/g/min, LnT2D = 2.16 [0.36] mL/g/min, O‐T2D = 1.60 [0.28] mL/g/min; *p* < 0.0001). There were no significant differences in rest MBF or in MPR between the four groups (Figure 3).

#### Qualitative assessment of myocardial perfusion and LGE imaging

Image quality for first‐pass perfusion was rated as good in all participants, with none of the participants demonstrating visual stress‐induced perfusion defects. None of the participants showed subendocardial hyperenhancement to indicate the presence of a chronic silent myocardial infarction.

#### VAT and SAT

**FIGURE 4:**
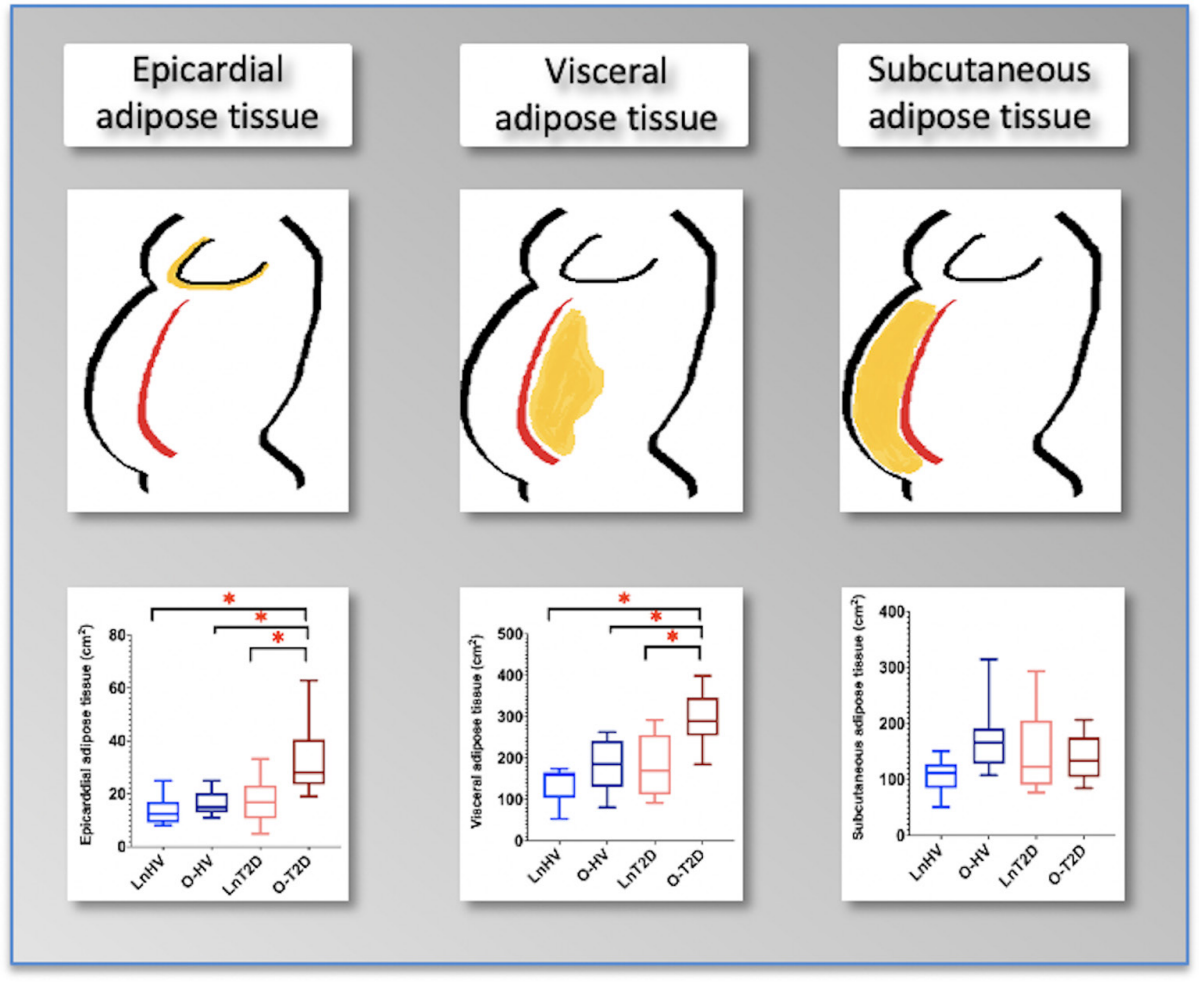
Box plot comparisons of epicardial, visceral, and subcutaneous adipose tissue. *p < 0.05. HV, healthy volunteers; LnHV, lean HV; LnT2D, lean patients with type 2 diabetes; O‐HV, HV with overweight; O‐T2D, patients with overweight and type 2 diabetes [Color figure can be viewed at wileyonlinelibrary.com]

Numerically, the LnHV group had the lowest SAT area (LnHV = 106 [31] cm^2^, O‐HV = 169 [55] cm^2^, LnT2D = 147 [71] cm^2^, O‐T2D = 171 [80] cm^2^; *p* = 0.06); however, this did not reach statistical significance. The VAT area was significantly higher in the O‐T2D group compared with the other three groups (LnHV = 127 [53] cm^2^, O‐HV = 181 [60] cm^2^, LnT2D = 182 [99] cm^2^, O‐T2D = 288 [72] cm^2^; *p* < 0.0001). This was also numerically increased in the O‐HV and LnT2D groups compared with the LnHV group, although it did not reach statistical significance (Figure 4). The patients in the O‐T2D group had the highest VAT‐SAT ratio; however, this was also significantly higher in patients in the LnT2D group compared with the control groups who were lean or overweight (LnHV and O‐HV groups).

#### EAT

The O‐T2D group had higher EAT area compared with the LnT2D, LnHV, and O‐HV groups (LnHV = 14 [6] cm^2^, O‐HV = 17 [5] cm^2^, LnT2D = 18 [8] cm^2^, O‐T2D = 31 [13] cm^2^; *p* < 0.0001).

#### Correlations between adipose tissue measurements

The correlations between metabolic parameters and visceral adiposity are provided in Table 3. There were significant positive correlations among all quantitative measures of adiposity (BMI and waist circumference, EAT, and VAT) among each of the measures.

**TABLE 3:** Correlations of BMI, VAT, EAT, and waist circumference.

	BMI		VAT		EAT		Waist circumference	
	*r*	*p* value	*r*	*p* value	*r*	*p* value	*r*	*p* value
BMI	–	–	0.47	**0.0002**	0.30	**0.01**	0.72	**<0.0001**
Waist circumference	0.72	**<0.0001**	0.76	**<0.0001**	0.63	**0.0002**	–	–
VAT	0.47	**0.0002**	–	–	0.73	**0.01**	0.76	**<0.0001**
EAT	0.30	**0.01**	0.73	**0.01**	–	**–**	0.63	**0.0002**

Note

Values in bold signify *p* < 0.05.

Abbreviations: EAT, epicardial adipose tissue; VAT, visceral adipose tissue.

#### Correlations of perfusion with functional parameters

The stress or rest MBF did not correlate with LV ejection fraction. There were significant but weak correlations of stress MBF with the following strain parameters: PDSR (*r* = 0.29, *p* = 0.03) and GLS (*r* = 0.30, *p* = 0.02).

#### Subgroup analyses

The study had inadequate power to assess subgroup analyses, and such tests were not planned. Nevertheless, the VAT data spread suggested heterogeneity within the LnT2D group, suggesting two groups of patients with LnT2D: those with VAT similar to the O‐T2D group and those similar to the LnHV group. When the LnT2D cohort was divided into two groups around the mean value for VAT (182 cm^2^), stress MBF was numerically higher in the participants with lower VAT; however, this trend did not reach statistical significance, likely because of small numbers (low VAT LnT2D stress MBF = 2.25 [0.40] mL/g/min vs. high VAT LnT2D stress MBF = 2.06 [0.30] mL/g/min; *p* = 0.3). When the O‐T2D cohort was divided into two groups around the mean value for VAT for this cohort (288 cm^2^), stress MBF was numerically higher in the participants with lower VAT. However, this trend again did not reach statistical significance (low VAT O‐T2D stress MBF = 1.59 [0.33] mL/g/min vs. high VAT O‐T2D stress MBF = 1.61 [0.20] mL/g/min; *p* = 0.8).

Moreover, when the two groups with T2D were subdivided based on their metformin treatment status, these subgroups showed no significant differences in any of the cardiac structural, functional, or perfusion parameters, except for the significant difference in circumferential strain in the O‐T2D group. Patients with O‐T2D who were receiving metformin treatment (metformin +, *n* = 15) exhibited significantly higher global circumferential strain (GCS) compared with 15 patients with O‐T2D who were not receiving metformin (GCS metformin [+] O‐T2D = −19.43% [1.79%] vs. GCS metformin [−] O‐T2D = −16.60% [3.84%]; *p* = 0.02). No such difference was detected in the LnT2D group based on metformin treatment status for any of the cardiac parameters.

## DISCUSSION

The results of the present study provide several new findings. First, myocardial stress perfusion was reduced only in the O‐T2D group, with no reduction in global stress MBF or MPR in the LnT2D and O‐HV groups compared with the LnHV group. Second, although the O‐T2D group showed greater visceral adiposity, accumulation of VAT was evident even in patients with T2D and normal body weight at levels similar to control patients with overweight and without diabetes. Third, in patients with T2D, all measures of adiposity strongly correlated with one another, and VAT, BMI, and waist circumference were each related to abnormalities in systolic and diastolic strain.

These results add further evidence for increased body weight as an important integrating determinant of myocardial perfusion in diabetes. Although capillary rarefaction has been proposed among the mechanisms of CMD in diabetes and obesity (35, 36), in this study, the reduction in stress MBF was detected in patients with O‐T2D despite no significant structural alterations, such as increases in LV mass, native T1, ECV, or myocardial cell volume calculations. We have not detected any association between insulin resistance (HOMA‐IR) and global rest or stress MBF or MPR. However, the O‐T2D group showed striking increases in visceral adiposity, with a 40% larger VAT area compared with the LnT2D group. The mechanistic link between increased body weight and impairment of the total vasodilator capacity might, therefore, include etiologies such as altered adipokine profile associated with VAT accumulation in patients with diabetes. Supporting this, adipokine profile in patients with T2D was shown in a previous study to depend on degree of adiposity, with no alterations in plasma adiponectin levels detected in the LnT2D group (37). The latter study also provided further evidence for significant associations between adipokine levels and plasma markers of systemic inflammation (37).

In a previous study, Sørensen and colleagues also explored the alterations in rest and stress MBF in patients with T2D (38) and also showed a significant reduction in stress MBF in the diabetes cohort compared with control patients. The investigators in that study included mainly patients with T2D who had overweight or obesity (mean BMI = 31.1 [4.6]), with a significant difference in the mean BMI compared with the control cohort (mean BMI = 25.3 [3.4]), which supports our finding that patients with T2D and overweight or obesity show significant alterations in myocardial perfusion. However, Sørensen and colleagues did not explore alterations in myocardial perfusion in patients with LnT2D or weight‐matched lean control patients with no diabetes and control patients with overweight and without diabetes. To our knowledge, ours is the only study addressing this specific question. Our study also differed in the perfusion analysis methodology. Whereas Sørensen and colleagues used an in‐house developed MATLAB tool for their manual perfusion analysis, the data in our study were analyzed via a machine‐learning algorithm developed and extensively validated by study collaborators (23, 39, 40). In this study, a deep neural network‐based computational workflow for inline myocardial perfusion analysis automatically delineated the myocardium. This computational neural network is capable of cardiac perfusion mapping and integrating an automated inline implementation on the MR scanner, enabling instant data analysis and reporting without manual assessment (39). These automated methods for MBF estimation from CMR investigations may soon provide new opportunities for screening of coronary microvascular disease in diabetes and obesity in routine clinical care.

The LV ejection fraction was significantly lower only in the O‐T2D group compared with the control groups, while still remaining within normal range. Moreover, systolic and diastolic strain parameters were also significantly lower only in the O‐T2D group. Among the global and segmental strain parameters, GLS has been shown to be the most reproducible strain parameter (41). Although not yet resolved, it is possible that the identification of subclinical LV dysfunction by the means of reductions in GLS may lead to management changes that will alter cardiac outcomes in obesity and diabetes (42).

Body composition analysis based on the characterization of different tissue compartments is increasingly used for both clinical and research questions. MRI, with its optimal soft tissue resolution and inherently high contrast between fat and water, is an ideal modality for the assessment of adipose tissue with high accuracy and precision and without the use of ionizing radiation. Neeland and colleagues previously assessed the associations of abdominal VAT and SAT mass on MRI with markers of cardiac and metabolic risk in a population‐based cohort of adults with obesity (43). They showed that VAT associated with an adverse metabolic, dyslipidemic, and atherogenic obesity phenotype, whereas SAT associated with a more benign phenotype, characterized by modest associations with inflammatory biomarkers and leptin, but no independent association with dyslipidemia, insulin resistance, or atherosclerosis in individuals with obesity. Although, in our study, VAT accumulation was also evident in patients with LnT2D, patients with O‐T2D exhibited the highest level of visceral adiposity among the study groups. In a longitudinal population‐based study, Kouli and colleagues confirmed the prognostic significance of excess visceral adiposity (44). They showed that visceral adiposity index derived from waist circumference, BMI, triglyceride, and HDL levels was independently associated with elevated 10‐year cardiovascular disease risk, particularly in men (44). In our study, in addition to the higher visceral adiposity, the O‐T2D cohort showed lower HDL levels, higher triglyceride levels, and higher waist circumference compared with patients in the LnT2D cohort or lean control patients with no diabetes and control patients who were overweight with no diabetes, suggesting a worse metabolic phenotype in the O‐T2D group.

Plasma adipokine levels or markers of systemic inflammation were not measured; however, large studies have previously reported the differences in adipokine profile and markers of systemic inflammation in patients who have T2D and who are lean or have obesity, which complemented our study by providing valuable insights for the interpretation of our findings (10, 37, 45).

A complete characterization of coronary microvascular function also requires assessment of the response to vasoconstrictor stimuli during an invasive coronary angiography procedure. Subjecting our participants to invasive coronary angiography was deemed unacceptable for asymptomatic patients and healthy control patients.

For the control cohort, at the point of recruitment, it was ascertained by verbal questioning that the exercise duration had been <6 h/wk for the past 12 months, which is similar to the cohorts with diabetes participating in this study. However, this was not objectively assessed by requesting participants to wear an exercise activity monitor in our attempt to minimize the overall burden of the study to participants. However, the control groups showed numerically higher cholesterol measurements and similar or higher systolic and diastolic BP measurements and resting heart rates, and they did not show any cardiac features that could be regarded as athlete’s heart, further confirming the point that these control patients were not uncommonly healthy and athletic individuals but represented the local retiree population with an activity level similar to the cohort with diabetes.

## CONCLUSION

Patients with T2D and normal body weight do not exhibit reductions in MBF, which suggests that increased body weight is an important integrating determinant of myocardial perfusion in patients with diabetes. Although patients with T2D and overweight showed greater visceral adiposity, accumulation of visceral fat is evident even in lean patients with T2D at levels similar to control patients with overweight and without diabetes.O

## CONFLICT OF INTEREST

The authors declared no conflict of interest.

## DATA AVAILABILITY STATEMENT

The data underlying this article will be shared on reasonable request to the corresponding author.
